# TRPA1 Is a Polyunsaturated Fatty Acid Sensor in Mammals

**DOI:** 10.1371/journal.pone.0038439

**Published:** 2012-06-19

**Authors:** Arianne L. Motter, Gerard P. Ahern

**Affiliations:** Department of Pharmacology and Physiology, Georgetown University, Washington, District of Columbia, United States of America; University of Houston, United States of America

## Abstract

Fatty acids can act as important signaling molecules regulating diverse physiological processes. Our understanding, however, of fatty acid signaling mechanisms and receptor targets remains incomplete. Here we show that Transient Receptor Potential Ankyrin 1 (TRPA1), a cation channel expressed in sensory neurons and gut tissues, functions as a sensor of polyunsaturated fatty acids (PUFAs) *in vitro* and *in vivo*. PUFAs, containing at least 18 carbon atoms and three unsaturated bonds, activate TRPA1 to excite primary sensory neurons and enteroendocrine cells. Moreover, behavioral aversion to PUFAs is absent in TRPA1-null mice. Further, sustained or repeated agonism with PUFAs leads to TRPA1 desensitization. PUFAs activate TRPA1 non-covalently and independently of known ligand binding domains located in the N-terminus and 5^th^ transmembrane region. PUFA sensitivity is restricted to mammalian (rodent and human) TRPA1 channels, as the drosophila and zebrafish TRPA1 orthologs do not respond to DHA. We propose that PUFA-sensing by mammalian TRPA1 may regulate pain and gastrointestinal functions.

## Introduction

Fatty acids are important signaling molecules and the detection of exogenous and endogenous fatty acids by nerves and other cells represents a fundamental physiological process. For example, taste receptor cells and enteroendocrine cells of the gastrointestinal system sense dietary fatty acids to regulate proper digestive processes and appetite [1]. Further, the sensing of phospholipid-derived fatty acids is a critical signaling pathway. Phospholipase A_2_ enzymes hydrolyze glycerophospholipids to release a free PUFA [2]. Additionally, diacyl glycerol (DAG) lipase may liberate a PUFA from DAG. In turn, PUFAs and their metabolites act on various targets to regulate inflammation, cell division and wound repair [3], [4].

Despite the importance of fatty acid signaling the molecular identity of critical fatty-acid sensors is unclear. Cluster of differentiation-36 (CD36) expressed in taste papillae binds saturated and unsaturated fatty acids and participates in orosensory-mediated digestive secretions [1]. Unsaturated fatty acids can also block delayed rectifier K^+^ channels to prolong depolarization of taste receptor cells [5]. Further, enteroendocrine tissue express several orphan GPCRs: GPR40 [6], GPR41 [7], [8], GPR43 [7], [8] and GPR120 [9] that are believed to participate in the sensing of saturated and unsaturated fatty acids. Finally, polyunsaturated fatty acids bind peroxisome proliferator-activated receptors to regulate gene expression [10].

Interestingly, Transient Receptor Potential (TRP) ion channels are candidate fatty acid sensors [11]. Phospholipase C signaling coupled to diacylgylcerol (DAG) activates *Drosophila* TRP and TRPL [11] as well as vertebrate TRPC 2, 3, 6 and 7 channels [12]. Further, polyunsaturated fatty acids (PUFAs), specifically linolenic acid, directly gate *Drosophila* TRP and TRPL [13] while 20-carbon PUFAs activate C. *elegans* TRPV channels [14]. Moreover, PUFAs can sensitize, activate or inhibit vertebrate TRP channels including TRPV1 [15], TRPV3 [16] and TRPM8 [17]. The precise physiological relevance of fatty-acid sensing by TRP channels is unclear.

Here we identify mammalian TRPA1 as a PUFA receptor. TRPA1, expressed primarily in nociceptive sensory neurons [18], detects a variety of noxious electrophilic compounds including mustard oil (allyl isothiocyanate, AITC) [19], [20], cinnamaldehyde [20], wasabi [19], and 4-hydroxynonenal, an endogenous unsaturated aldehyde [21]. In addition, several chemicals activate TRPA1 in a non-covalent fashion including icilin [22], menthol [23], and noxious general anesthetics [24]. Of note, disruption of TRPA1 expression attenuates pain-hypersensitivity indicating an important role for TRPA1 in nociception [18]. In addition, TRPA1 expression in various gut tissues (intrinsic enteric neurons [25], inhibitory motor neurons [26] and enteroendocrine cells [27], [28], [29]) suggests novel, non-nociceptive functions for TRPA1 that remain to be elucidated. We show that TRPA1 mediates PUFA- induced excitation of sensory neurons and enteroendocrine cells, as well as PUFA-induced aversive behaviors in mice. PUFAs directly activate, in a non-covalent manner, mammalian TRPA1 but do not activate the drosophila nor zebrafish channel orthologs. These data, establish mammalian TRPA1 as a PUFA detector both *in vitro* and *in vivo*.

## Results

### TRPA1 is Critical for Sensing PUFAs in Sensory Neurons

**Figure 1:**
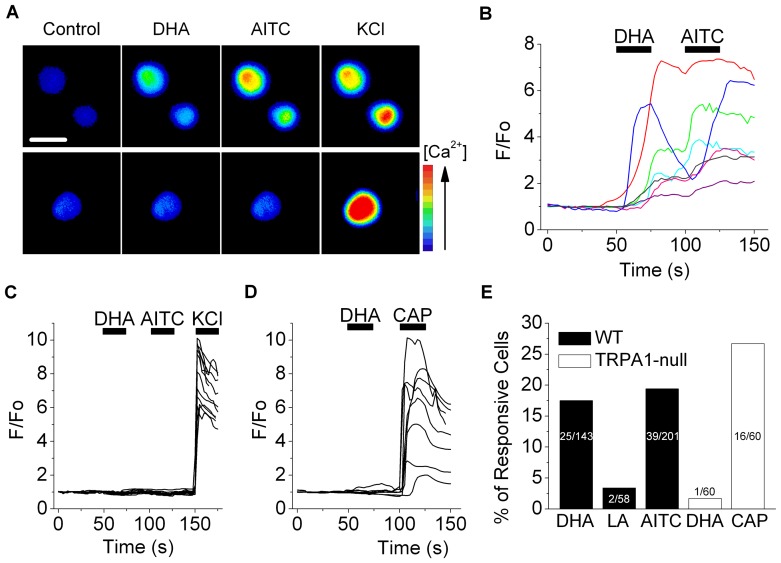
TRPA1 is critical for sensory nerve detection of PUFAs. (A) Fluo-4 fluorescence images of DHA-responsive neurons (top panel) and a non-responsive neuron (bottom panel). Scale bar is 40 µm. (B&C) Representative Ca^2+^ transients evoked by DHA (100 µM), AITC (1 mM), KCl (140 mM) in DRG neurons (*B*, DHA responsive; *C*, DHA-unresponsive). (D) Ca^2+^ transients evoked by DHA (100 µM) or capsaicin (CAP, 100 nM) in DRG neurons from TRPA1-null mice. (E) The percentage of WT or TRPA1-null neurons responsive to DHA (WT, n = 25 of 143; TRPA1-null, n = 1 of 60), LA (WT, n = 2 of 58), AITC (WT, n = 39 of 201) or CAP (TRPA1-null, n = 16 of 60).

In our search for novel fatty acid receptors we were guided by the observation that PUFAs have an aversive quality both in worms [14] and mammals [30]. To explore whether this property arises from a direct activation of nociceptors, we performed Ca^2+^ imaging in cultured, mouse sensory neurons. Figure 1A&E shows that docosahexaenoic acid (DHA, C22∶6), increased intracellular [Ca^2+^] in a subset (17.5%, 25 of 143 cells) of sensory neurons. In contrast, the saturated fatty acid, lauric acid (LA,C12∶0), elicited little response (3.4%, 2 of 58 cells; Fig. 1E and Fig. S1). Strikingly, these DHA-sensitive neurons all responded to AITC, an agonist of the TRPA1 channel (Fig. 1B). In contrast, no responses to DHA were observed in AITC-insensitive neurons (Fig. 1C). To test a critical role for TRPA1 in sensing DHA, we examined sensory neurons derived from TRPA1-deficient mice. Figure 1D&E shows that DHA-evoked responses were mostly absent in these cells (1.7%, 1 of 60 cells), whereas these TRPA1-null cells displayed sensitivity to capsaicin (26.7%, 16 of 60 cells), a ligand for the TRPV1 channel that is commonly co-expressed with TRPA1 [18]. Thus, TRPA1 appears critical for sensory nerves to detect PUFAs. Although we showed previously that PUFAs activate TRPV1 [15], this agonistic effect required concomitant protein kinase C-mediated phosphorylation that sensitizes TRPV1 to various agonists. Under resting non-inflammatory conditions, therefore, TRPA1 is the predominant sensor of PUFAs.

### TRPA1 Mediates Taste-aversion to PUFAs in Mice

**Figure 2:**
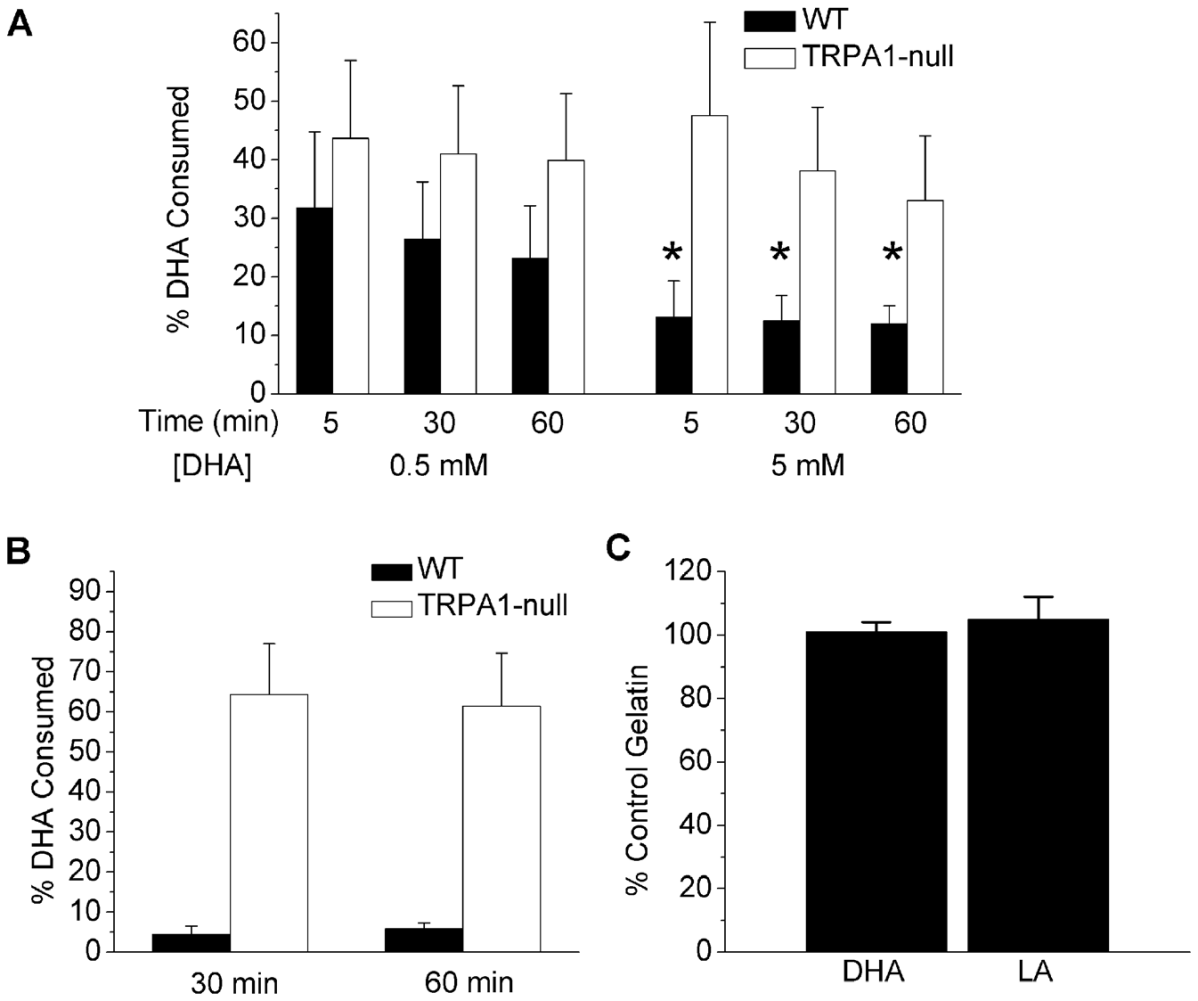
TRPA1 mediates taste aversion to PUFAs. (A) Percentage consumption of gelatin with or without DHA (0.5 or 5 mM) in a two-taste preference test by wild-type (n = 10) or TRPA1-null mice (n = 7). *p<0.05 T-test. (B) Percentage consumption of DHA (5 mM) or LA (5 mM, n = 3) in a two-taste preference test. (C) Percentage of DHA (5 mM) or LA (5 mM) consumed in a single taste test compared to lipid-free gelatin consumed the previous day (wild-type only, n = 4).

To test whether TRPA1 contributes to oral aversion to fatty acids we performed two-taste preference tests. Mice trained to eat a sweetened, flavored gelatin, were presented with gelatin supplemented with or without DHA (0.5 and 5 mM). Figure 2A shows that wild-type mice showed a negative preference to DHA at 5 mM, whereas TRPA1-mice in contrast, displayed no preference. Although fatty acid texture could contribute to taste preferences, we found that wild-type mice preferred lauric acid to DHA in a two-taste preference test (Fig. 2B), arguing against non-specific textural effects of DHA. Further, if given no choice, mice consumed similar amounts of gelatin with or without DHA (Fig. 2C) arguing against strict avoidance of DHA. Thus, TRPA1 appears essential for the sensory nerve detection of PUFAs in vivo and accompanying behavioral responses.

### PUFAs Activate Mammalian TRPA1 Channels

**Figure 3:**
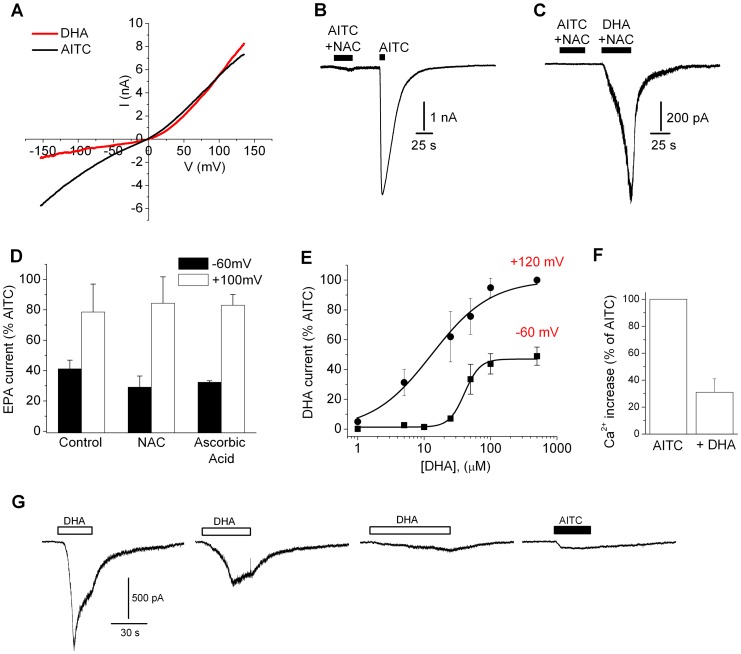
DHA activates rat TRPA1 in transfected HEK293 cells. (A) Representative *I–V* relationship for DHA (100 µM) and AITC (1 mM) in a voltage-clamped TRPA1-expressing HEK293 cell. (B&C) N-acetyl-L-cysteine (NAC, 15 mM) prevents the activation of TRPA1 current by AITC (1 mM) but not DHA (100 µM). (D) Normalized EPA-evoked currents (% of 1 mM AITC) in control buffer (n = 4), NAC (15 mM, n = 4), or Ascorbic Acid (15 mM, n = 4). (E) DHA activates TRPA1 currents in a dose- and voltage-dependent manner (n = 3–5; holding potential −60 mV or +120 mV). The smooth lines show the best-fits to a Hill equation yielding an E_max_ of 49±6%, EC_50_ of 41±5 µM and Hill Coefficient of 3.8±1.3 for −60 mV, and E_max_ of 100%, EC_50_ of 13.2±1.8 µM and Hill Coefficient of 1.0±0.1 for +120 mV. (F) Normalized increase in intracellular [Ca^2+^] evoked by AITC in TRPA1-expressing HEK293 cells with or without pre-treatment with DHA (100 µM, 10 min, n = 40–50). DHA was washed out for 2 min prior to AITC challenge. (G) Representative currents evoked by repeated application of DHA (100 µM) and AITC (500 µM) in a voltage-clamped TRPA1-expressing HEK293 cell.

To explore if DHA directly activates TRPA1 we performed voltage-clamp experiments in TRPA1-expressing HEK293 cells. Figure 3A shows that DHA (100 µM) evoked an outwardly-rectified current that reversed near 0 mV, consistent with activation of TRPA1. Immediate application of AITC (1 mM) to this cell enhanced the current at negative potentials, generating a voltage-insensitive response typical of a high concentration of agonist [31]. In contrast, DHA failed to evoke currents in mock-transfected cells (n = 3). Potentially, activation of TRPA1 could involve DHA itself, or a DHA metabolite. Indeed, unsaturated fatty acids can oxidize in aqueous solutions to form electrophiles, and electrophilic compounds can covalently modify and activate TRPA1 [22], [32]. Therefore, to exclude an electrophilic effect we co-applied the anti-oxidant N-acetyl-L-cysteine (NAC). Figure 3B shows that co-application of NAC (15 mM) fully suppressed activation by the TRPA1 agonist, AITC, demonstrating that NAC can effectively sequester electrophiles under these conditions. In contrast, DHA and eicosapentaenoic acid (EPA, C20∶5, 100 µM) evoked robust currents in the presence of NAC or ascorbic acid (Figs. 3C&D) indicating unambiguously that these PUFAs, and not oxidized products, stimulate TRPA1. Concentration-response analysis for DHA (at −60 mV, Fig. 3E) revealed an EC_50_ of 41 µM with an efficacy of ∼50%. Interestingly, membrane depolarization to +120 mV increased the efficacy and potency to 100% and 9 µM respectively, consistent with the ability of TRPA1 [23] and other thermosensitive TRP channels to synergistically integrate physical (voltage) and chemical stimuli [31], [33]. Thus, DHA is a partial agonist at resting membrane potentials that can synergize with other TRPA1 stimuli.

TRPA1 is well known to undergo Ca^2+^-dependent desensitization. As seen with other agonists, we found that DHA-evoked currents exhibited a rapid component of decay during DHA application and also a reduction in the peak responses with repeated application (tachyphylaxis) (Fig. 3G). Interestingly, repeated DHA applications largely inhibited subsequent responses to AITC (500 µM, Fig. 3G.) To quantify this effect, we measured AITC-evoked Ca^2+^ responses in HEK293 cells with or without pretreatment with DHA. Figure 3F shows that DHA (100 µM for 10 minutes) reduced the response to AITC by ∼70%. We also found that pre-treatment with AITC (500 µM) completely inhibited subsequent responses to DHA (100 µM) in voltage-clamped HEK293 cells (−60 mV, n = 4).

**Figure 4:**
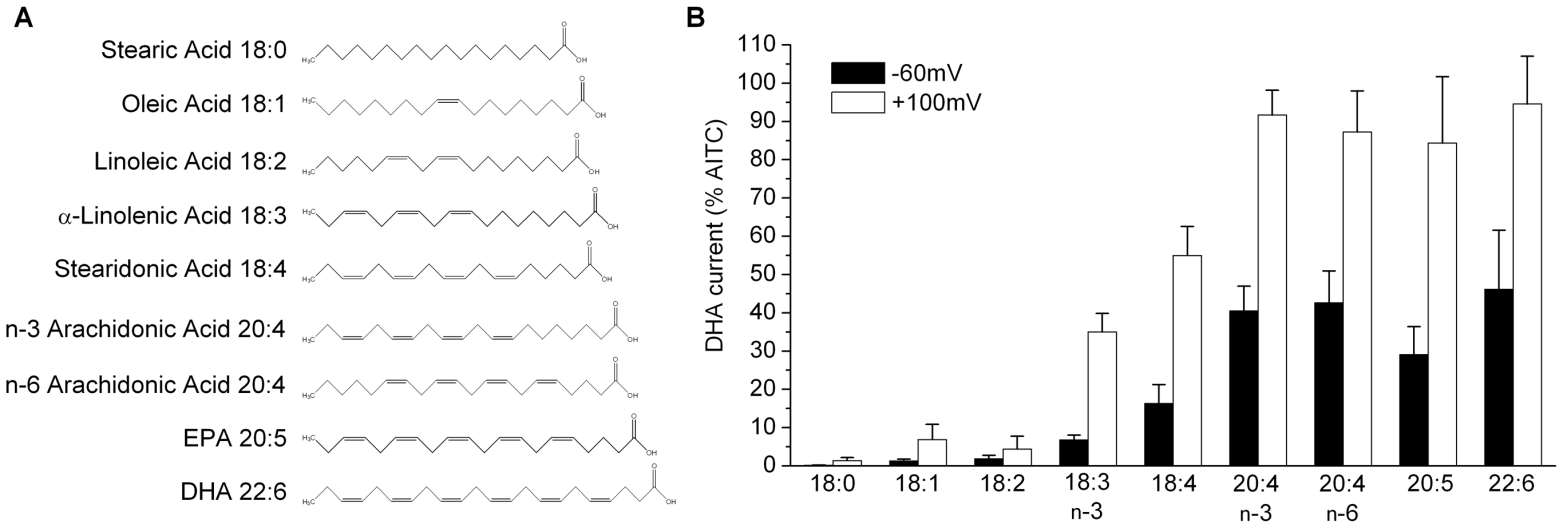
Activation of TRPA1 depends on fatty acid chain length and unsaturation. (A) Structures of fatty acids. (B) Normalized currents (% of 1 mM AITC) evoked by fatty acids (100 µM) at −60 or +100 mV in HEK293 cells transfected with rat TRPA1 (n = 4–8).

Next, we investigated the structural requirements for fatty acid activation of TRPA1 by comparing effects of fatty acids with varying carbon chain length and unsaturation. Figure 4 shows that activation of TRPA1 requires the presence of at least three double bonds, and maximal activity is obtained with four double bonds and twenty carbon atoms. Additionally, n−3 and n−6 PUFAs produced equivalent activation.

**Figure 5:**
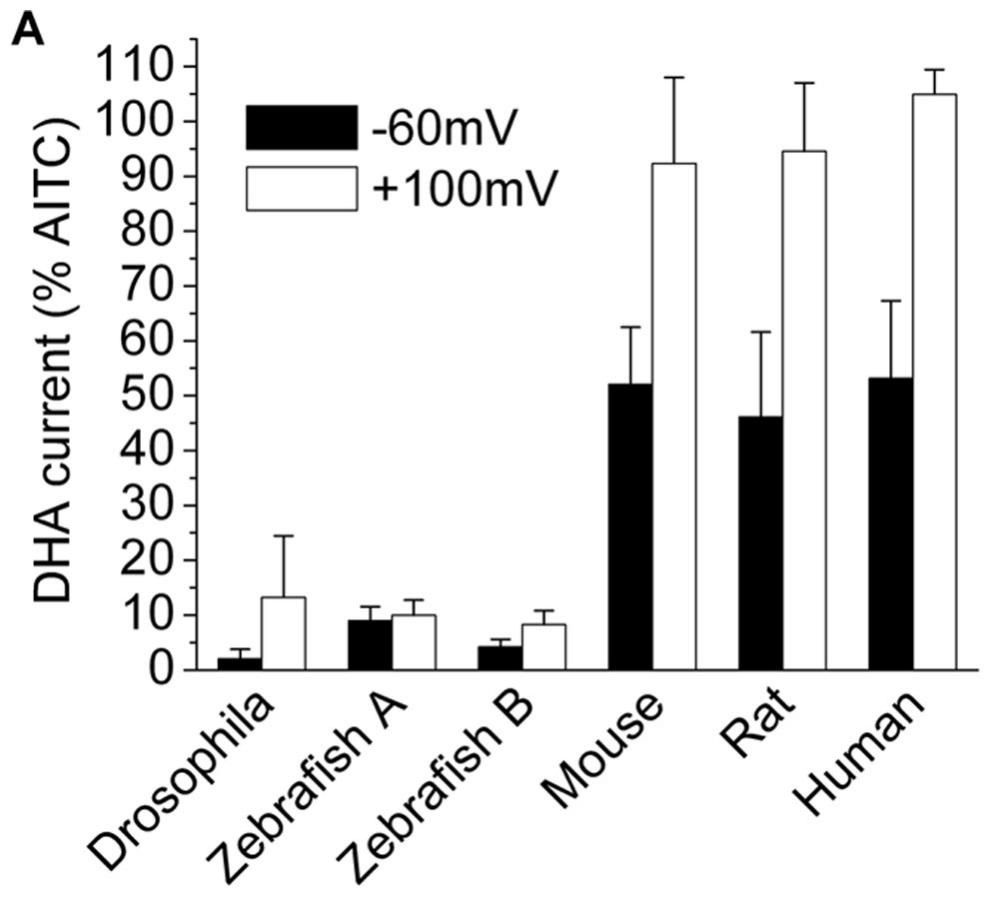
DHA selectively activates mammalian TRPA1 channels. (A) Normalized responses to 100 µM DHA at −60 and +100 mV in HEK293 cells transfected with either drosophila, zebrafish A, zebrafish B, mouse, rat or human TRPA1 (n = 3–6).

The nociceptive function of TRPA1 is remarkably conserved across animal species; insects to humans employ TRPA1 as a sensor of harmful electrophiles [34]. We therefore asked if fatty acid sensing is similarly conserved. Figure 5 and Fig. S2 show that DHA (100 µM) robustly activates mouse, rat and human TRPA1, with maximal efficacy at potentials greater than +100 mV. Surprisingly, and in contrast, DHA (100 µM) evoked little to no activation of drosophila or zebrafish (Zebrafish A&B) TRPA1 at both negative and positive potentials. This concentration of DHA is ∼8–10-fold higher than the EC_50_ value measured in rat (+120 mV see Fig. 3E). Thus, fatty acid sensing appears to be selective for mammalian TRPA1 channels.

### PUFAs Activate TRPA1 via an Intracellular-accessible Domain and Independently of the N-terminus and Transmembrane 5

Next, we explored potential mechanisms for PUFA activation of TRPA1. First, we considered whether or not PUFAs act on the plasma membrane and independently of the TRPA1 protein. Indeed, unsaturated fatty acids can increase membrane fluidity/elasticity [35] and this property could potentially underlie activation of TRPA1. However, we found that the micelle-forming detergent and membrane fluidizer, Triton X-100 (50 µM) [36], produced negligible activation of TRPA1 at negative or positive potentials (0.3±0.2% and 1±1% of AITC at −60 mV and +100 mV respectively, n = 3), arguing against membrane fluidity effects.

**Figure 6:**
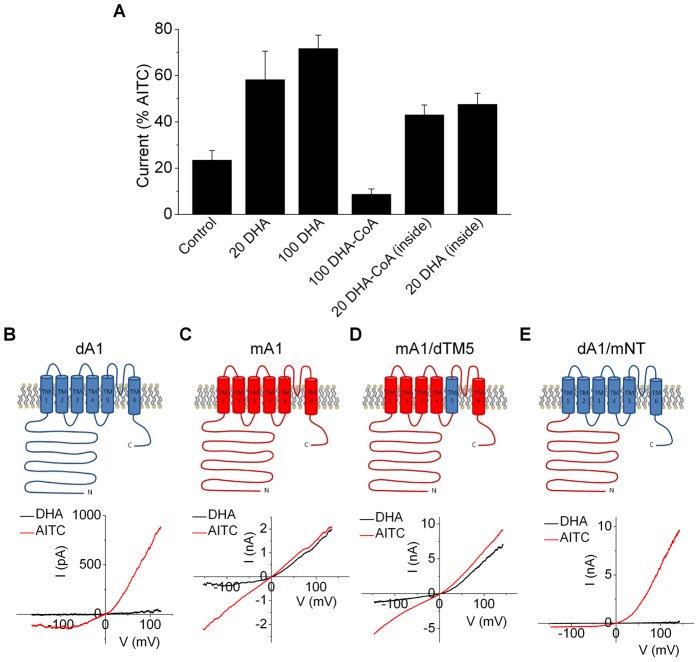
PUFAs activate TRPA1 independently of the N-terminus or transmembrane domain 5. **A.** Mean current responses in rTRPA1-expressing HEK293 cells (+100 mV; n = 3–4) for control, extracellular DHA (20 µM or 100 µM) and DHA-CoA (100 µM), or intracellular application of DHA-CoA (20 µM) and DHA (20 µM). The background current is not subtracted. (B–E) *I–V* relationship for responses to DHA (100 µM) and AITC (1 mM) in HEK293 cells expressing chimeric drosophila-mouse TRPA1 channels.

Next, we explored for regions in TRPA1 necessary for fatty acid agonism. To discriminate between an extracellular and intracellular mode of action, we compared effects of the coenzyme A derivative of DHA, DHA-CoA. The addition of coenzyme A increases the hydrophilicity of the fatty acid, effectively rendering it membrane impermeable. Figure 6A shows that extracellular application of DHA-CoA failed to activate TRPA1. In contrast, intracellular application of DHA-CoA (by inclusion in the patch pipette solution) evoked currents similar to DHA. These data indicate that DHA engages TRPA1 at the cytoplasmic face or in the inner leaflet of the membrane. Structure-function studies have revealed two broad domains required for ligand activation of TRPA1. The N-terminus contains several cysteines and a lysine essential for activation by electrophiles [22], [32]. In addition, residues in the 5^th^ transmembrane domain (TM5) mediate sensitivity to the non-covalent ligand, menthol [37]. To test a role for these domains in fatty-acid sensing we studied TRPA1 chimeras composed of drosophila (DHA-insensitive) and mouse (DHA-sensitive) proteins (Figs. 6B–E). Figure 6D shows that substituting the mouse N-terminus fails to confer DHA-sensitivity to dTRPA1, although the chimera responds robustly to AITC. Thus, the N-terminus alone is not sufficient for detecting DHA. Further, exchanging mouse TM5 with the dTM5 did not affect sensitivity to DHA (Fig. 6E). Thus, neither the N-terminus nor TM5 appear to be directly involved in fatty acid sensing.

### DHA Activates TRPA1 and Secretion in Enteroendocrine Cells

**Figure 7:**
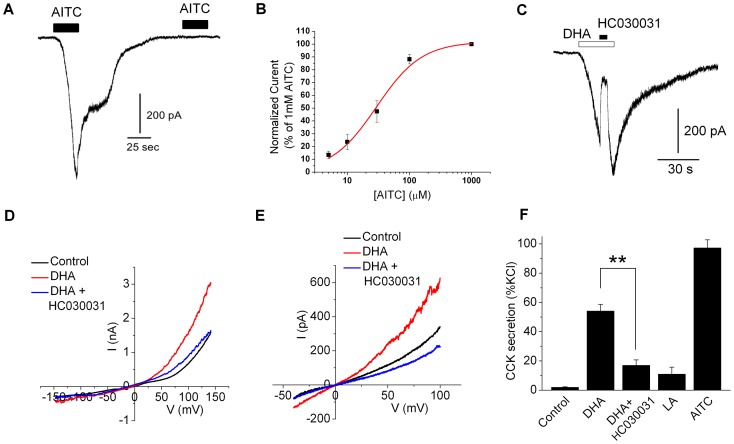
DHA activates TRPA1 and secretion in enteroendocrine cells. (A) Representative current trace showing response of voltage-clamped STC-1 cell to successive applications of AITC (500 µM). (B) Concentration-response curve for AITC evoked currents in STC-1 cells (−60 mV, n = 3–4 for each point). The smooth lines show the best-fit to a Hill equation yielding an EC_50_ of 29±3 µM and Hill Coefficient of 1.2±0.1 (C) DHA (100 µM) activates an inward current in an STC-1 cell that is blocked reversibly by HC030031 (20 µM). (D) *I–V* relationship in a voltage-clamped STC-1 cell demonstrating response to DHA (100 µM) and block by the TRPA1 antagonist, HC030031 (50 µM). (E) *I–V* relationship for DHA (100 µM) with and without HC030031 (50 µM) in a voltage-clamped RIN14B cell. (F) CCK secretion from STC-1 cells stimulated by 100 µM DHA, DHA plus 50 µM HC030031, 500 µM LA, 100 µM AITC and 140 mM KCl (n = 4–12) T-test **p<0.01.

Interestingly, in addition to sensory nerves, TRPA1 is found in enteroendocrine cells of the mammalian gut. *In situ* hybridization identifies TRPA1 in epithelial cells facing the gut lumen [27]. In particular, enterochromaffin cells (ECs) [27], the EC-like cells lines, RIN14B [27] and QGP-1 [28], and the I-cell line STC-1 [29] highly express TRPA1. We therefore tested effects of AITC and DHA in several EC cell lines (obtained from American Type Culture Collection). Electrophysiological measurements of TRPA1 in STC-1 cells have not been reported previously. Therefore we first confirmed TRPA1 functionality by measuring AITC-sensitivity in voltage-clamped cells. Figure 7A shows that AITC activates an inward current (−60 mV) and this response exhibits characteristic desensitization. Concentration-response analysis (Fig. 7B) revealed an EC_50_ of 29 µM and Hill slope of 1.2, similar to values previously reported for rodent TRPA1 expressed heterologously (11–33 µM [19], [20]). Next, we examined the sensitivity of EC cells to PUFAs. Figure 7C–E shows that DHA activates an outwardly-rectified conductance in voltage-clamped STC-1 and RIN14B cells, that is inhibited by the TRPA1 antagonist, HC030031. Finally we measured secretion of the gut hormone, cholecystokinin (CCK). Figure 7F shows that the TRPA1 agonists, DHA and AITC evoke robust secretion of CCK from STC-1 cells. In contrast, the saturated fatty acid, LA, elicits a significantly smaller level of release. Notably, DHA-evoked CCK release was inhibited by HC030031 (Fig. 7F). In addition, we found that pre-treatment with AITC to functionally ablate TRPA1 responses decreased CCK secretion by 57±12% (n = 3, P<0.01). In these experiments we pre-applied AITC in the presence of ruthenium red (10 µM) to avoid Ca^2+^-triggered depletion of CCK, and we found that AITC did not affect the maximal depolarization-evoked CCK release which was ∼20-fold under control or pre-treated conditions. Taken together, these data show that TRPA1 confers sensitivity to PUFAs in enteroendocrine cells and direct activation of TRPA1 by DHA contributes to CCK secretion.

## Discussion

Our data identify mammalian TRPA1 as a molecular detector of PUFAs. Although PUFAs are known to modulate several TRP channels the precise physiological functions of these effects are unclear. We show that PUFAs activate TRPA1 and that TRPA1 is essential for PUFAs to excite sensory neurons and enteroendocrine cells. Moreover, behavioral aversion to oral ingestion of PUFAs requires TRPA1 and is defective in TRPA1-null mice.

Comparative analysis of fatty acid structures showed that both the level of unsaturation and carbon-chain length determine fatty acid activity at TRPA1. Activation of TRPA1 requires a minimum of three double bonds and maximum efficacy occurs with four double bonds and twenty carbon atoms. Thus, AA, EPA and DHA possess approximate equivalent efficacy. DHA, unlike AA and EPA, is poorly metabolized and therefore these data support a direct action of fatty acids rather than PUFA metabolites. This is consistent with an earlier Ca^2+^ imaging study showing that both AA and a non-metabolizable AA congener stimulate TRPA1 expressed in HEK293 cells [20]. Unsaturated fatty acids are susceptible to auto-oxidation resulting in the formation of various electrophilic derivatives [38] that could potentially explain activation of TRPA1. In addition, nitrated fatty acids containing a nitroalkene group potently activate TRPA1 [39], [40], while oxidized linoleic acid metabolites activate TRPV1 [41]. However, we found that PUFAs retained activity even in the presence of high concentrations of NAC that is effective in fully sequestering electrophiles such as AITC. PUFAs are membrane fluidizers [35], [36] and increased membrane fluidity could therefore account for opening of TRPA1. We observed, however, that another membrane fluidizer, Triton-X 100 exerted little effect on TRPA1 activity. Furthermore, increasing the hydrophilicity, and thereby restricting the membrane penetrance, of DHA with addition of a Coenzyme A group did not abolish PUFA agonism, arguing against a non-specific effect on membrane properties. Thus, the most parsimonious explanation is that PUFAs act directly on the TRPA1 protein. Interestingly, PUFAs also modulate TRPV1 activity, and competitively displace [^3^H]-resiniferatoxin binding, suggesting that PUFAs directly interact with TRPV1 [15].

The results of previous studies have identified two domains in TRPA1 involved in ligand sensing. The N-terminus contains several cysteines and a lysine required for covalent modification by electrophiles [22], [32]. Interestingly, thermal sensing in snake TRPA1 may also reside in the N-terminal ankyrin repeat domains [42]. Outside of the N-terminus the fifth transmembrane domain (TM5) is implicated in menthol- and thymol-sensing [37]. Surprisingly, our analysis using chimeric drosophila-mouse TRPA1 channels suggests that neither the N-terminus nor TM5 is directly involved in sensing PUFAs. Rather these data suggest that other transmembrane or intracellular domains contain PUFA interaction sites. However, we can not exclude the possibility that the N-terminus acts in concert with these other regions. Unfortunately, the non-functionality of many of the mammalian-drosophila chimeras [37] restricted further exploration of putative PUFA-sensing domains. Nonetheless, the species-specific differences in PUFA activation of TRPA1 may prove useful in determining critical sites required for PUFA sensing. Interestingly, lipoxygenase-generated DHA derivatives, termed resolvins, are reported to be TRPA1 antagonists [43]. Resolvin D1 blocks TRPA1 in a non-competitive fashion [43], and potentially PUFAs and their derivatives act at the same site to affect channel gating in a positive or negative manner.

Although the precise physiological role of PUFA-sensing by TRPA1 is unclear, our data suggest potential roles in nociceptive signaling and/or gut function. The generation of PUFAs such as AA following stimulation of PLA_2_ activity is a fundamental signaling pathway in inflammation and pain. Activated cytosolic PLA_2_ hydrolyzes the sn-2 position of glycerophospholipids to release AA and lysophospholipid. Further, diacylglycerol lipase can liberate AA from diacylglycerol. In turn, this AA may directly activate TRPA1, in addition to being metabolized by cyclooxygenase, lipoxygenase or P450 enzymes into algesic products and electrophilic TRPA1 agonists [44], [45]. Although the absolute sensitivity of PUFA activation at TRPA1 appears low (EC_50_ of 41 µM at −60 mV), it is on par with that reported for the prostaglandin agonists [44], [45]. In addition, it is important to note that this EC_50_ reflects extracellular application of fatty acid and the effective biologic concentration may be much lower. Indeed, PUFAs likely act at the cytoplasmic face based on the efficacy of intracellular DHA-CoA. Thus, in sensory neurons, near-membrane microdomains of AA may be sufficient to effectively activate TRPA1. In addition, PUFAs may act in concert with other ligands, for example, cytoplasmic Ca^2+^ [19], [46], [47], [48] and voltage [23] that enhance PUFA potency and/or efficacy at TRPA1. Indeed, we observed that that membrane depolarization increases efficacy to 100%. Interestingly, Ca^2+^ is a key activator of cPLA_2_. Therefore, an increase in cytoplasmic [Ca^2+^] (a weak TRPA1 stimulus) may synchronize with the generation of AA to produce a greater TRPA1 response. On the otherhand, we show that sustained DHA application leads to desensitization of TRPA1, raising the possibility that PUFAs may exert a predominant analgesic action. In addition, agonism of TRPA1 by PUFAs (that notably occurs with slow kinetics) may potentially induce analgesia via depolarization block, as demonstrated for metabolites of acetaminophen [49].

Exogenous sources of PUFAs may also be relevant to nociception. PUFAs, although abundant in the diet, are normally consumed in the form of triglycerides. However, high concentrations of free fatty acids can accumulate in spoiled or rancid food. Indeed, free PUFAs, as well as oxidized PUFAs, contribute to the unpleasant “fishy” taste of spoiled fish [30]. Our data indicate that taste aversion to PUFAs occurs via TRPA1. Presumably this is mediated by activation of TRPA1-expressing trigeminal nerves that innervate the oral cavity. Similarly, caffeine produces taste aversion in rodents through activating TRPA1 [50]. In contrast, PUFAs produce aversive behavior in C. *elegans* via TRPV channels [14], consistent with the worm TRPA1 lacking PUFA sensitivity.

Most dietary fatty acids are liberated from triglycerides in the small intestine by pancreatic lipases, generating high concentrations of FFAs and monoacylglycerols in the gut lumen. Enteroendocrine cells facing the gut lumen, detect these FFAs and other nutrients and in turn secrete gut hormones such as 5-HT and CCK that regulate gut motility, gut secretions, gallbladder contraction and gastric emptying [51], [52]. Notably, enteroendocrine cells express functional TRPA1 channels that can trigger secretion of CCK [29] or 5-HT [27]. In turn, TRPA1-mediated secretion of 5-HT induces smooth muscle contraction [27] and modulates gastric emptying [28]. We show that PUFAs activate TRPA1 in enteroendocrine cell lines and evoke CCK secretion. Thus, TRPA1 could function as a gut nutrient sensor, detecting PUFAs to elicit appropriate hormonal responses. In this regard, it is interesting to note that mammals are obligate consumers of C20–22 dietary PUFAs during early post-natal development. Neonates have impaired capacity to synthesize PUFAs (by desaturating and elongating linoleic acid) [53], [54], and therefore preferentially obtain AA, EPA and DHA directly from breast milk. Neonates also have less efficient fat absorption [55]. Therefore, it would be interesting to assess a role for TRPA1 in PUFA-sensing in the neonate gut. For example, PUFA-sensing by TRPA1-expressing enteroendocrine cells could prolong gastrointestinal transit time, an effect that would be adaptive in neonates. In addition to enteroendocrine cells, intrinsic enteric neurons [25], inhibitory motor neurons [26] and gut projecting primary afferent neurons (DRG and vagal) [56], [57] all express TRPA1, but it is doubtful that these are relevant targets for dietary PUFAs.

In summary, our data show that mammalian TRPA1 acts as a discrete sensor of long-chain PUFAs and TRPA1 confers cellular and behavioral sensitivity to PUFAs.

## Materials and Methods

All experimental procedures involving animals were approved by the Georgetown University Animal Care and Use Committee (#07–124) and conform to NIH guidelines.

### Ca^2+^ Imaging

Dorsal root ganglia (DRG) were cultured from adult mice (mixed background B6/129P wild-type and TRPA1-null (B6;129P-*Trpa1^tm1Kykw^*/J), Jackson Labs, 12–16 weeks) in Neurobasal supplemented with 2% B-27 medium (Invitrogen), 0.1% L-glutamine and 1% penicillin/streptomycin. Neurons were loaded with 1 µM Fluo4-AM (Invitrogen) for 20 min and washed for another 10–20 min before recording. The dye was excited at 480±15 nm. Emitted fluorescence was filtered with a 535±25 nm bandpass filter, captured by a SPOT RT digital camera (Diagnostic Instruments) and read into a computer. Analysis was performed offline using Simple PCI software (Compix Inc.).

### Electrophysiology

HEK 293F cells (obtained from American Type Culture Collection) were transfected with drosophila TRPA1 (gift of Paul Garrity, Brandeis University), zebrafish A and B TRPA1 (gift of Alex Schier, Harvard University), mouse TRPA1 (gift of Jaime García-Añoveros, Northwestern University), rat TRPA1 (gift of David Julius, University of California San Francisco), human TRPA1 (gift of Sven Jordt, Yale University), or chimeric drosophila-mouse TRPA1 (gift of Ardem Patapoutian, The Scripps Research Institute). STC-1 cells (from American Type Culture Collection) were cultured in high glucose DMEM (Hyclone) supplemented with 15% horse serum, 2.5% FBS, 1% L-glutamine, 1% penicillin/streptomycin. RIN14B cells (from American Type Culture Collection) were cultured in high glucose RPMI 1640 supplemented with 10% FBS, 1% L-glutamine and 1% penicillin/streptomycin. Whole cell and single-channel patch-clamp recordings were performed using an EPC8 amplifier (HEKA Electronics). For whole-cell recordings the bath solution contained 140 mM NaCl, 4 mM KCl, 1 mM MgCl_2_, 1.2 mM CaCl_2_, 10 mM HEPES, 5.6 mM glucose, pH 7.3. 15 mM N-acetyl cysteine was added to bath solutions containing DHA. The pipette solution contained 140 mM NaCl or CsCl, 10 mM HEPES, 5 mM EGTA, pH 7.3. Solutions were applied via a gravity-fed system and separate outlets were used to apply fatty acids, capsaicin and AITC solutions to prevent cross-contamination. Concentration-response data were measured by application of DHA followed immediately by a saturating concentration of AITC, and data were normalized to AITC. Current-voltage measurements consisted of a 150-ms ramp from −150 mV to +150 mV for transfected HEK293 and STC-1 cells. For RIN14B cells we used a 1 second pre-pulse to +70 mV to desensitize sodium channels prior to a 150-ms ramp from −50 mV to +150 mV. Unless otherwise stated the baseline currents under control conditions were subtracted.

### CCK Secretion

STC-1 were plated on 35×10 mm plates and cultured for 2–3 days. Cells were washed twice with 1 ml HBBS and incubated for 20 min at room temperature with HBBS containing stimulants (625 ml/well). Supernatants were collected and spun to remove cell debris. CCK was detected using an EIA kit (Phoenix Pharmaceutical). Stimulated cells were lysed and analyzed for total protein using a Bradford assay. Peptide secretion was normalized to total protein for each plate.

### Behavioral Tests

Mice were housed in groups and had access to food and water *ad libitum*. Male and female were individually placed in an empty cage and allowed to acclimate for 30 min before testing. Prior to the testing day, mice were trained to eat gelatin from two dishes within 60 min. Only mice that consumed equally from both dishes were included. The gelatin contained 10% (w/v) Polycose (Ross Nutrition/Abbot), 2.5% (w/v) gelatin (Knox/Kraft Foods), 2.5% (w/v) sucrose, and 0.04% (v/v) orange flavoring (McCormick & Co., Inc.). On testing day the gelatin was supplemented with fatty acids at varying concentrations based on Cartoni et al. [58]. We used flavored gelatin to minimize effects of fatty acid texture and smell.

### Fatty acids and Chemicals

Capsaicin (CAP) and HC030031 were obtained from Tocris Bioscience (Ellisville, MO, USA). Allyl isothiocyanate (AITC), stearic and lauric acid were purchased from Sigma-Aldrich. Oleic, linoleic α-linolenic, stearidonic, ω-3 and ω-6 arachidonic acids, DHA, and EPA with 0.1% BHT, were obtained from Cayman Chemical (Ann Arbor, MI, USA). Stock solutions of drugs were prepared in ethanol and diluted to physiological solution prior to the experiments; final ethanol concentration was always <0.1%, which had no effect on TRPA1 activity. Fatty acids are readily oxidized in aqueous solutions therefore, the antioxidant, N-acetyl L-cysteine (15 mM), was added and fresh solutions were made every 20 min. In some experiments BSA (0.01%) was added to improve fatty acid solubility.

## Supporting Information

Figure S1 **Saturated fatty acids do not activate sensory neurons.** (A&B) Lauric acid (LA, 100 µM) evokes little change in [Ca^2+^] in both AITC (1 mM)-sensitive and AITC-insensitive DRG neurons cultured from wild-type mice.
(TIF)

Figure S2 **Current-voltage relationships for DHA and AITC in drosophila, zebrafish and mammalian TRPA1 channels.** (A–D) I–V relationship for DHA (100 µM) and AITC (1 mM) responses in HEK293 cells transfected with drosophila, zebrafish A, zebrafish B, mouse, rat, or human TRPA1.
(TIF)
