# Supplementary figures and images for: TRPA1 Is a Polyunsaturated Fatty Acid Sensor in Mammals
Source: PLoS One. 2012 Jun 19;7(6):e38439. doi: 10.1371/journal.pone.0038439 (PMC3378573; doi:10.1371/journal.pone.0038439)

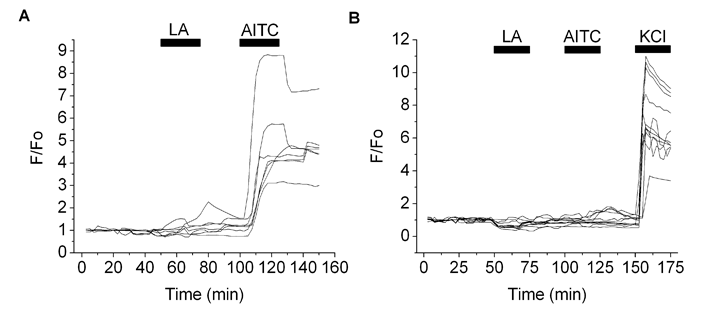

Supplement: Figure S1 — Saturated fatty acids do not activate sensory neurons. (A&B) Lauric acid (LA, 100 µM) evokes little change in [Ca2+] in both AITC (1 mM)-sensitive and AITC-insensitive DRG neurons cultured from wild-type mice. (TIF) [file pone.0038439.s001.tif]

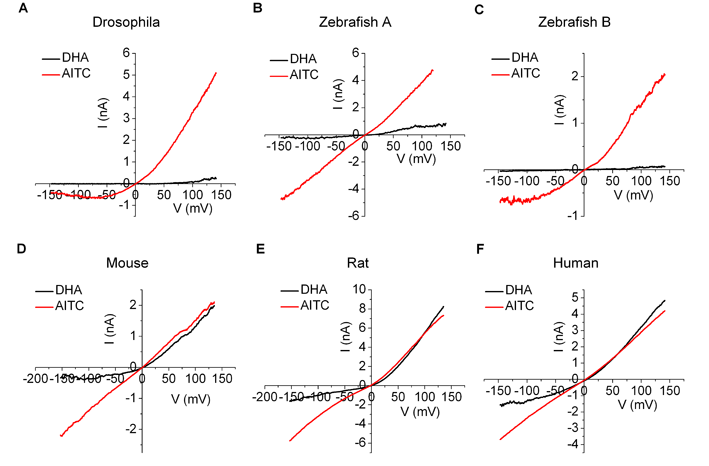

Supplement: Figure S2 — Current-voltage relationships for DHA and AITC in drosophila, zebrafish and mammalian TRPA1 channels. (A–D) I–V relationship for DHA (100 µM) and AITC (1 mM) responses in HEK293 cells transfected with drosophila, zebrafish A, zebrafish B, mouse, rat, or human TRPA1. (TIF) [file pone.0038439.s002.tif]
